# A Novel SiC Trench MOSFET with Self-Aligned N-Type Ion Implantation Technique

**DOI:** 10.3390/mi14122212

**Published:** 2023-12-07

**Authors:** Baozhu Wang, Hongyi Xu, Na Ren, Hengyu Wang, Kai Huang, Kuang Sheng

**Affiliations:** 1 School of Micro-Nano Electronics, Zhejiang University, Hangzhou 310027, China; wbz@zju.edu.cn (B.W.); huangk@zju.edu.cn (K.H.); 2 ZJU-Hangzhou Global Scientific and Technological Innovation Center, Hangzhou 311200, China; ren_na@zju.edu.cn (N.R.); shengk@zju.edu.cn (K.S.); 3 College of Electrical Engineering, Zhejiang University, Hangzhou 310027, China; wanghengyu@zju.edu.cn

**Keywords:** silicon carbide (SiC), trench MOSFET, self-aligned, P-epi layer, oxide protection

## Abstract

We propose a novel silicon carbide (SiC) self-aligned N-type ion implanted trench MOSFET (NITMOS) device. The maximum electric field in the gate oxide could be effectively reduced to below 3 MV/cm with the introduction of the P-epi layer below the trench. The P-epi layer is partially counter-doped by a self-aligned N-type ion implantation process, resulting in a relatively low specific on-resistance (R_on,sp_). The lateral spacing between the trench sidewall and N-implanted region (W_sp_) plays a crucial role in determining the performance of the SiC NITMOS device, which is comprehensively studied through the numerical simulation. With the W_sp_ increasing, the SiC NITMOS device demonstrates a better short-circuit capability owing to the reduced saturation current. The gate-to-drain capacitance (C_gd_) and gate-to-drain charge (Q_gd_) are also investigated. It is observed that both C_gd_ and Q_gd_ decrease as the W_sp_ increases, owing to the enhanced screen effect. Compared to the SiC double-trench MOSFET device, the optimal SiC NITMOS device exhibits a 79% reduction in C_gd_, a 38% decrease in Q_gd_, and a 41% reduction in Q_gd_ × R_on,sp_. A higher switching speed and a lower switching loss can be achieved using the proposed structure.

## 1. Introduction

Silicon carbide (SiC) power devices show great potential in high-voltage and high-temperature power electronics applications due to their superior material properties, such as a wide band gap, high critical electric field, high electron saturation velocity and high thermal conductivity [1,2]. SiC MOSFET devices can realize higher switching speeds and lower switching losses than the conventional Si power devices and have been widely applied in electric vehicles [3,4,5]. However, SiC planar MOSFET devices still have relatively high specific on-resistance (R_on,sp_) owing to their low channel mobility [6,7]. On the other hand, SiC trench MOSFET (TMOS) devices are expected to realize a lower R_on,sp_ owing to the absence of the JFET region, smaller cell pitch and higher channel mobility on the trench sidewall [8]. However, during the blocking state, SiC trench MOSFET devices suffer from a high electric field in the gate oxide at the trench corner, which can result in long-term reliability issues [9].

The P+ shield regions are commonly introduced in SiC trench MOSFET devices to alleviate the electric field crowding at the trench corner. In recent years, various structural enhancements have been implemented in SiC trench MOSFET devices to address the aforementioned issues, such as SiC double trench MOSFET (DTMOS) [10], SiC implantation-and-epitaxial trench MOSFET (IETMOS) [11], SiC asymmetrical cell trench MOSFET (ATMOS) [12], SiC trench MOSFET with multiple-ion-implantation into tilted trench side walls (MIT^2^-MOS) [13] and Fin-SiC TMOS [14]. The fabrication process of the SiC DTMOS device includes an extra step of etching the source trench, followed by a vertical self-aligned P-type ion implantation to form the P+ shield region. In addition, a tilted P-type ion implantation into the sidewalls of the source trench is utilized to achieve a short connection between the P+ shield region and the source. The SiC IETMOS device requires two additional P-type ion implantations and two additional epi-layer regrowth steps to create the P+ shield region. In the SiC MIT^2^-MOS device, the P+ shield region can be simply formed through a vertical self-aligned P-type ion implantation into the bottom of the gate trench. However, an additional tilted Al implantation is required for grounding, which will reduce the current conducting area of the device. The P+ shield regions in the SiC ATMOS and Fin-SiC TMOS devices are formed only through ultra-high energy (MeV) ion implantation. However, this process results in the introduction of more defects that need to be repaired and makes it challenging to control the shape of the implanted region, which is crucial for device performance. Therefore, to simplify and improve the fabrication process, an easy-to-fabricate structure is required.

In this work, a novel self-aligned N-type ion implanted SiC trench MOSFET (NITMOS) device is proposed and studied. The device features P-type regions located below the trench, partially overlapping with both sides of the trench bottom in the vertical direction. The presence of these P-type regions introduces an undesirable JFET region, which can be optimized to achieve a low R_on,sp_ and maximum electric field in the gate oxide at the same time. In addition, the short circuit capability and gate-to-drain capacitance are investigated using numerical simulations. 

## 2. Device Structure and Parameters

**Figure 1:**
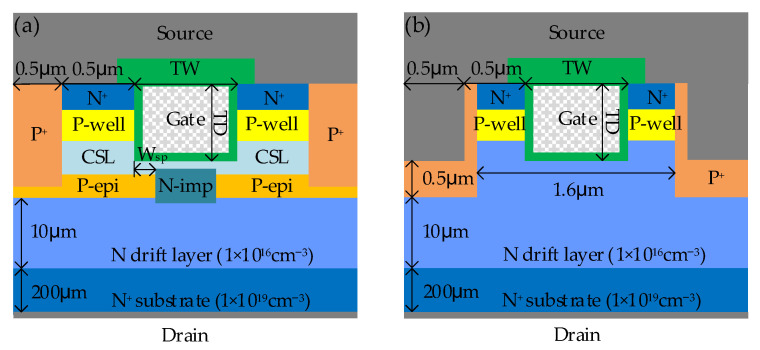
Schematic cross sections and key parameters of (**a**) SiC NITMOS and (**b**) SiC DTMOS cell structures.

Figure 1 shows the schematic cross sections of SiC NITMOS and SiC DTMOS devices. A P-type epitaxial layer (P-epi in Figure 1a) grown on top of the N drift layer was utilized to protect the gate oxide at the trench bottom. The proposed SiC NITMOS device was based on the self-aligned N-type implantation technology to incorporate N-type regions (N-imp in Figure 1a), and conduct currents through the P-epi layer. The lateral spacing between the trench sidewall and N-imp (W_sp_ in Figure 1a) can be precisely controlled through a self-alignment process. A relatively heavy doping current spreading layer (CSL in Figure 1a) was introduced to decrease the depletion width caused by the P-epi layer and expand the current conducting region. The P+ region was utilized to achieve a short connection between the P-epi layer and the source. Compared to the floating P-type shield region, the grounded P-type shield region can effectively reduce the maximum electric field in the gate oxide, increase the switching speed of the device and improve the short-circuit capability of the device, which contributes to enhancing the device performance and reliability [15,16,17]. Considering the current fabrication capabilities, the trench width (TW in Figure 1a), trench depth (TD in Figure 1a), and cell pitch of the device were set to 1 μm, 1 μm and 3 μm, respectively. The channel had a length of 0.4 μm and an electron mobility of 40 cm^2^/V·s [18,19]. Furthermore, the thickness of the gate oxide was 50 nm at the sidewall and bottom of the trench. The simulation parameters are summarized in Table 1. 

In this work, Silvaco was utilized to conduct both device and mixed-mode simulations. In the simulation, the electron/hole continuity and Poisson equations were solved self-consistently, considering the Shockley–Read–Hall recombination, carrier generation, complete dopant ionization, electron/hole mobility, electron/hole saturation velocity, and impact ionization. The key physical models are specified below:

(1) The mobility model utilized in our analysis takes into consideration the impact of doping concentration, electric field, and temperature on the carrier mobility. This model accurately represents the behavior of carrier transport within the device. The low field mobilities, which are dependent on doping concentration and temperature, can be described using the following analytical function, based on the work by Caughey and Thomas [20]:

$$\mu_{n0,p0}=\mu_{n,p}^{min}{\left(\frac{T}{300\;K}\right)}^{\alpha_{n,p}}+\frac{\mu_{n,p}^{max}{\left(\frac{T}{300\;K}\right)}^{\beta_{n,p}}-\mu_{n,p}^{min}{\left(\frac{T}{300\;K}\right)}^{\alpha_{n,p}}}{1+{\left(\frac{T}{300\;K}\right)}^{\gamma_{n,p}}{\left(\frac{N}{N_{n,p}^{crit}}\right)}^{\delta_{n,p}}}$$

where *N* is the local impurity concentration in cm^−3^ and *T* is the temperature in degrees Kelvin. $N_{n}^{crit}\;$ = 1.94 × 10^17^ cm^−3^ and $N_{p}^{crit}\;$ = 1.76 × 10^19^ cm^−3^. While $\mu_{n}^{min}\;$ = 0 cm^2^/V·s, $\mu_{p}^{min}\;$ = 15.9 cm^2^/V·s, $\mu_{n}^{max}$ = 947 cm^2^/V·s and $\mu_{p}^{max}$ = 124 cm^2^/V·s. In addition, the coefficients $\alpha_{n,p}$, $\beta_{n,p}$, $\gamma_{n,p}$, and $\delta_{n,p}$ are fitting coefficients used in the model.

(2) The saturation effect on carrier drift velocity under high electric fields is taken into account, which accurately accounts for the limitation of carrier velocity in the device and its influence on overall device performance. As carriers are accelerated in an electric field, their velocity will saturate when the electric field magnitude reaches a significant level. The field-dependent mobility is implemented using the following Caughey and Thomas Expression [20]. This expression provides a smooth transition between low-field and high-field behavior, effectively describing the carrier behavior under various electric field conditions.

$$\mu_{n,p}\left(E\right)=\frac{\mu_{n0,p0}}{{\left(1+{\left(\frac{\mu_{n0,p0}E}{\nu_{sat}}\right)}^{\beta_{E}}\right)}^{\frac{1}{\beta_{E}}}}$$

where *E* is the parallel electric field, $\beta_{E}$ is the fitting coefficient, and $\mu_{n0}$ and $\mu_{p0}$ are the low field electron and hole mobilities, respectively. In addition, the saturation velocity ($\nu_{sat}$) is approximately 2.0 × 10^7^ cm/s for both electrons and holes.

(3) The impact ionization model used in the simulation was proposed by S. Selberherr [21], which is based on the following equation:

$$\alpha_{n,p}(E)=A_{n,p}exp\left[-{\left(\frac{B_{n,p}}{E}\right)}^{\beta_{n,p}}\right]$$

where $A_{n,p}$, $B_{n,p}$ and $\beta_{n,p}$ are fitting coefficients. The values of these parameters were selected by fitting experimental results presented by A. O. Konstantinov [22]. The impact ionization coefficients **α*_n_* and **α*_p_* as a function of electric field *E* can be expressed as

$$\alpha_{n}(E)=7.26\times 10^{6}\cdot exp\left[-\frac{2.34\times 10^{7}}{E}\right]{cm}^{-1}$$

$$\alpha_{n}(E)=7.26\times 10^{6}\cdot exp\left[-\frac{2.34\times 10^{7}}{E}\right]{cm}^{-1}$$

These simulation models and parameters utilized in this work have also been applied in other research conducted by our group, which includes various devices such as SiC junction barrier Schottky diodes (JBS), SiC trenched junction barrier Schottky diodes (TJBS), SiC planar MOSFET devices, SiC Super Junction Schottky diodes (SJ SBD) and SiC Super Junction JFET devices (SJ JFET) [23,24,25,26]. The simulated results of those devices are in good agreement with the experimental results obtained from fabricated devices.

## 3. Device Simulation and Results Discussion

**Figure 2:**
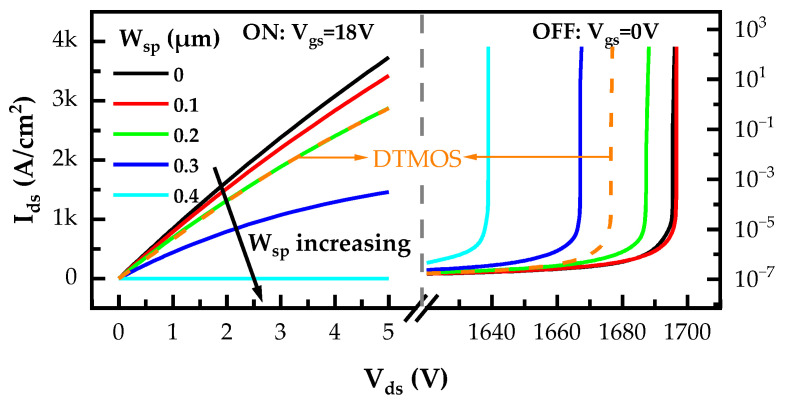
Simulated output and blocking characteristics of SiC DTMOS and SiC NITMOS devices with varying W_sp_.

The W_sp_ is of great significance to the SiC NITMOS device performance. Considering the trench width of 1 μm, the SiC NITMOS devices with a W_sp_ varying from 0 μm to 0.4 μm were studied. The simulated output and blocking characteristics of SiC DTMOS and SiC NITMOS devices with varying W_sp_ are shown in Figure 2. These results show that with the W_sp_ increasing from 0 μm to 0.4 μm, the SiC NITMOS device exhibited a progressive degradation in current conduction capability and reached a nearly non-conductive state at W_sp_ = 0.4 μm. The SiC DTMOS device showed a comparable conducting current to that of the SiC NITMOS device, with W_sp_ = 0.2 μm. 

**Figure 3:**
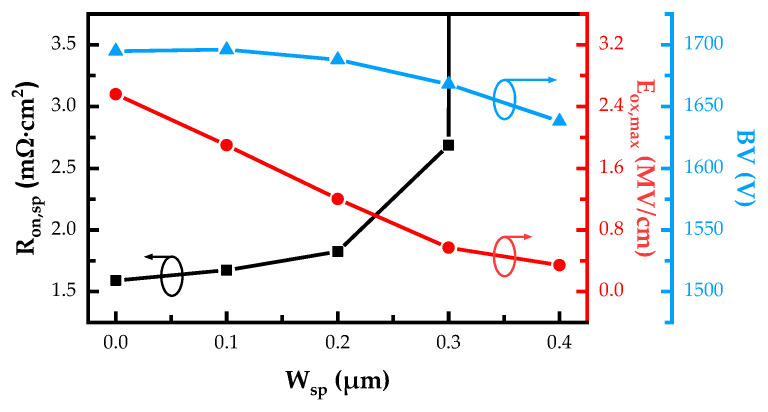
Simulated R_on,sp_, E_ox,max_ and BV of the SiC NITMOS devices with varying W_sp_.

**Figure 4:**
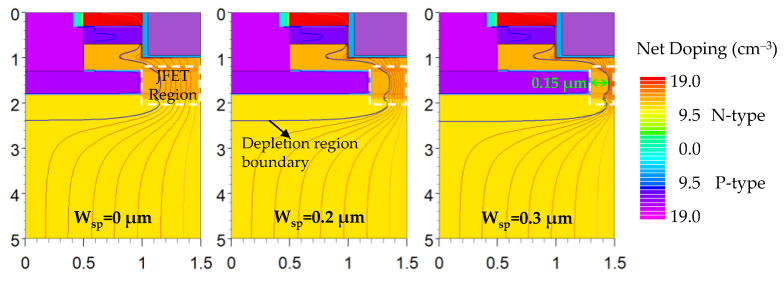
Simulated depletion region boundary and current flowline distributions of the SiC NITMOS devices with W_sp_ = 0, 0.2 and 0.3 μm at V_gs_ = 18 V and V_ds_ = 1 V.

**Figure 5:**
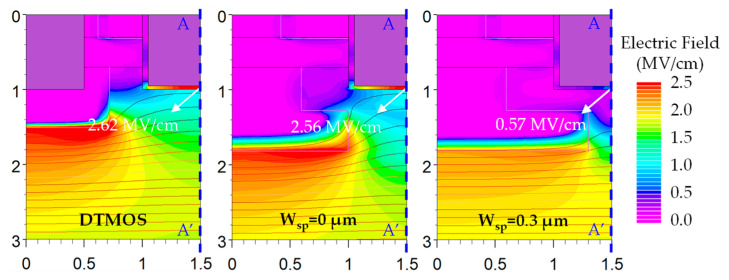
Simulated electric field and equal potential line distributions of SiC DTMOS and SiC NITMOS devices with W_sp_ = 0 μm and 0.3 μm at V_gs_ = 0 V and V_ds_ = 1200 V. The step of equal potential lines is 20 V.

**Figure 6:**
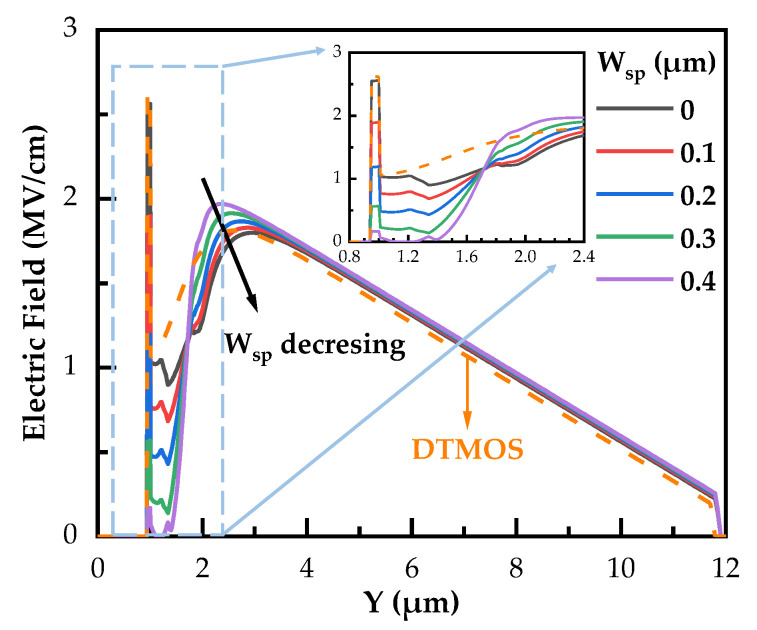
Simulated electric field distributions along A–A’ of SiC DTMOS and SiC NITMOS devices with varying W_sp_.

The specific on-resistances (R_on,sp_), breakdown voltages (BV) and maximum electric fields in the gate oxide (E_ox,max_) of the SiC NITMOS devices with varying W_sp_ are plotted in Figure 3. The R_on,sp_ of these simulated structures were extracted at V_ds_ = 1 V and the breakdown voltages were extracted at a leakage current density of 10^–3^ A/cm^2^ from their I_ds_–V_ds_ characteristics, where the avalanche breakdown in the device had already occurred. When the W_sp_ increased from 0 μm to 0.3 μm, the R_on,sp_ of the SiC NITMOS devices increased from 1.59 mΩ·cm^2^ to 2.69 mΩ·cm^2^. When the W_sp_ exceeded 0.3 μm, the device exhibited a sharp and substantial increase in the specific on-resistance. Such increments are attributed to the reduction in the width of the JFET region (the region inside the white dashed box in Figure 4) with the increasing W_sp_, as shown in Figure 4. As the W_sp_ increased, the BV initially increased slightly and then decreased, reaching its maximum value of 1696 V at W_sp_ = 0.1 μm. The E_ox,max_ was obtained at a blocking voltage of 1200 V and demonstrated a linear decrease with increasing W_sp_. The impact of the W_sp_ on the E_ox,max_ was explored using the electric field analysis, as shown in Figure 5 and Figure 6.

The simulated depletion region boundary and current flowline distributions of the SiC NITMOS devices with W_sp_ = 0, 0.2 and 0.3 μm at a gate-source voltage of 18 V and a drain-source voltage of 1 V are displayed in Figure 4. As the W_sp_ increased, the width of the JFET region decreased, resulting in increased crowding of the conducting current and an increase in the specific on-resistance. This effect was particularly pronounced when the W_sp_ increased from 0.2 μm to 0.3 μm, as shown in Figure 3. The maximum depletion width in the N-imp region induced by the P-epi layer was 0.15 μm. This implies that when the W_sp_ exceeds 0.35 μm, the adjacent depletions will merge, resulting in a loss of the device’s current conducting capability.

In order to ensure the long-term reliability of the device, it is generally recommended to maintain the E_ox,max_ below 3 MV/cm [27,28,29]. The simulated electric field and equal potential line distributions of SiC DTMOS and SiC NITMOS devices with W_sp_ = 0 μm and 0.3 μm at a gate-source voltage of 0 V and a drain-source voltage of 1200 V are depicted in Figure 5. It can be observed that the E_ox,max_ of SiC DTMOS and SiC NITMOS devices are located at the middle of the trench bottom, rather than at the corner of the trench bottom [10]. The E_ox,max_ of the SiC NITMOS device is much lower than 3 MV/cm. With the presence of the P-epi layer, the potential lines are prevented from passing through the N-imp region, mitigating the electric field crowding at the trench bottom. By increasing the W_sp_ from 0 μm to 0.3 μm, a significant reduction in the E_ox,max_ is observed, decreasing from 2.56 MV/cm to 0.57 MV/cm. Such a reduction ensures the good long-term reliability of the gate oxide [30]. The E_ox,max_ of the SiC DTMOS device was 2.62 MV/cm, which was slightly higher than that of the SiC NITMOS device with W_sp_ = 0 μm. Figure 6 shows the electric field distributions along A–A’ of the SiC DTMOS and SiC NITMOS devices with varying W_sp_ in Figure 5. With the W_sp_ increasing from 0 μm to 0.4 μm, the electric field at y = 2.4 μm experienced an increase, while the electric field at y = 1.3 μm and near the trench bottom was gradually reduced to 0 MV/cm. In addition, in the region between y = 3 μm and y = 12 μm, the electric field of the SiC DTMOS device was slightly lower than that of the SiC NITMOS device. However, near the trench bottom, the electric field of the SiC DTMOS device was significantly higher compared to the SiC NITMOS device, as shown in the inset of Figure 6. Such an observation indicates that the SiC NITMOS device shows a more effective shielding effect. This shielding effect in the SiC NITMOS device, induced by the P-epi layer, exhibits a comparable ability to reduce the electric field near the trench bottom. This effect can significantly mitigate electric field crowding, resulting in an improved device performance and reliability.

**Figure 7:**
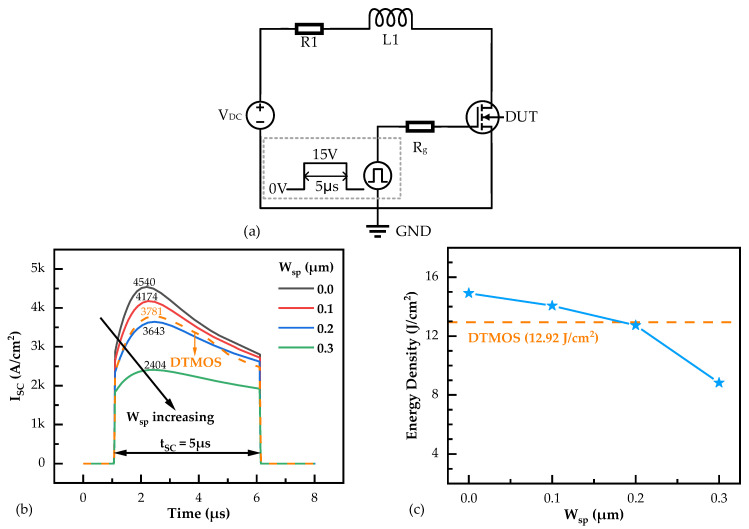
(**a**) The test circuit for the short circuit used in the simulation. (**b**) Drain current densities of SiC DTMOS and SiC NITMOS devices with varying W_sp_ under the short-circuit conditions at a DC voltage of 800 V. (**c**) Energy dissipations of SiC DTMOS and SiC NITMOS devices with varying W_sp_ in the short circuits at a DC voltage of 800 V.

In addition, the short-circuit capabilities of the SiC DTMOS and SiC NITMOS devices were estimated using the test circuit illustrated in Figure 7a. The DC voltage (V_DC_) was 800 V and the pulse width, i.e., the short circuit duration time (t_SC_), was 5 μs. The simulated short-circuit test waveforms of the SiC DTMOS and SiC NITMOS devices with varying W_sp_ are presented in Figure 7b. With the W_sp_ increasing from 0 μm to 0.3 μm, the maximum short-circuit current density (I_SC,max_) of the SiC NITMOS devices decreased from 4540 A/cm^2^ to 2404 A/cm^2^. This decrease is attributed to the reduced saturation current, as shown in Figure 2. The I_SC,max_ of the SiC DTMOS device was 3781 A/cm^2^, which was slightly higher than that of the SiC NITMOS with W_sp_ = 0.2 μm. The energy dissipations of the SiC DTMOS and SiC NITMOS devices with varying W_sp_ in the short circuit tests were also calculated, as shown in Figure 7c. As the W_sp_ increased from 0 μm to 0.3 μm, the generated energy density of the SiC NITMOS device decreased from 14.92 J/cm^2^ to 8.82 J/cm^2^. The lower energy density in the SiC NITMOS device resulted in reduced heat dissipation during the short-circuit period. This reduction indicates that the SiC NITMOS device with a larger W_sp_ possesses a better short-circuit capability and a longer short-circuit withstand time [31,32,33].

**Figure 8:**
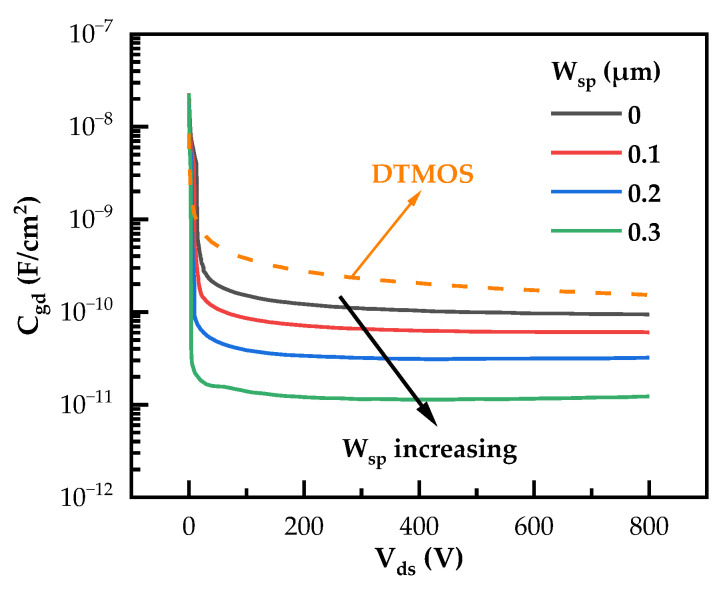
C_gd_–V_ds_ curves of SiC DTMOS and SiC NITMOS devices with varying W_sp_.

**Figure 9:**
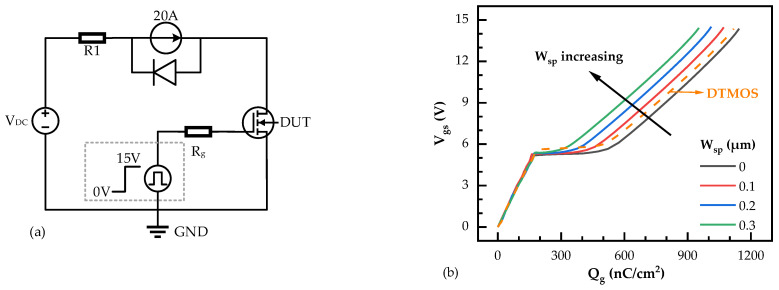
(**a**) The test circuit for the gate charges used in the simulation. The DC voltage was 800 V during the test. (**b**) Gate charges versus gate-source voltage for SiC DTMOS and SiC NITMOS devices with varying W_sp_.

The W_sp_ also plays a critical role in influencing the gate-to-drain capacitance (C_gd_) of the SiC NITMOS devices, which has a significant impact on the switching performance [34,35,36]. The C_gd_ values of SiC DTMOS and SiC NITMOS devices with varying W_sp_ are plotted in Figure 8. The SiC DTMOS device exhibited the highest C_gd_ during high-drain-source voltage conditions compared to the SiC NITMOS devices. This is because the gate was completely exposed to the drain in the SiC DTMOS device. The SiC NITMOS device exhibited a reduced C_gd_ as the W_sp_ increased. This is due to the enhanced screen effect, which reduced the coupling between the gate and drain, resulting in a lower C_gd_. The gate charges of the studied devices were evaluated using the test circuit shown in Figure 9a. The DC voltage was 800 V and an ideal diode was used as the freewheeling diode. As can be seen from Figure 9b, the Q_gd_ of the SiC DTMOS device was 323 nC/cm^2^, which was slightly lower than that of the SiC NITMOS device with W_sp_ = 0 μm. This can be attributed to the fact that the SiC DTMOS device had lower C_gd_ in the range of V_ds_ < 15 V. For the SiC NITMOS devices, as the W_sp_ increased from 0 μm to 0.3 μm, the Q_gd_ decreased from 361 nC/cm^2^ to 123 nC/cm^2^, which is significantly lower than that in the SiC DTMOS device.

The key characteristics of the SiC DTMOS and SiC NITMOS devices are summarized in Table 2 for comparison. Among the SiC NITMOS devices, the device with W_sp_ = 0 μm demonstrated a superior Baliga’s Figure of Merit (BFOM). However, the device also had the highest values for E_ox,max_, C_gd_ and Q_gd_ × R_on,sp_. On the other hand, the devices with W_sp_ = 0.2 μm and 0.3 μm demonstrated lower E_ox,max_, and similar Q_gd_ × R_on,sp_. In addition, the device with W_sp_ = 0.2 μm exhibited a better BFOM. Taking all factors into consideration, the device with W_sp_ = 0.2 μm seems to have the best structure. Compared to the SiC DTMOS device, the SiC NITMOS device with W_sp_ = 0.2 μm showed significant improvements, with a 79% reduction in C_gd_, a 38% decrease in Q_gd_, and a 41% reduction in Q_gd_ × R_on,sp_. Such significant reductions can result in higher switching speeds and lower switching losses.

## 4. Device Fabrication Process

**Figure 10:**
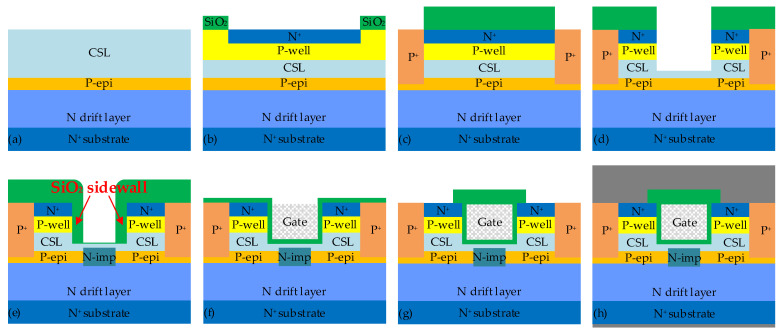
The proposed fabrication process for the SiC NITMOS device. (**a**) Starting wafer (**b**) P-well and N+ implantation (**c**) P+ implantation (**d**) Trench etching (**e**) N-imp implantation (**f**) Gate formation (**g**) Source region opening (**h**) Source and Drain electrodes formation.

A simple and practical fabrication process for the SiC NITMOS device is provided in Figure 10. The fabrication process begins with an N+ substrate and three epitaxial layers, including the N drift, P-epi and CSL layers (Figure 10a). The P-well and N+ regions are sequentially formed by a series of ion implantation steps (Figure 10b). The P+ region is implanted deeply into the P-epi layer to establish a short connection between the P-epi layer and the source (Figure 10c). The gate trench is etched using an Inductively Coupled Plasma (ICP) etching system with a SiO_2_ mask (Figure 10d). Subsequently, a SiO_2_ layer is deposited without removing the etch mask, and followed by an overall etchback process. The remaining SiO_2_ sidewall serves as a mask for self-aligned N-type ion implantation to create the N-imp region (Figure 10e), which is a critical process in the overall fabrication of the SiC NITMOS device. This process is of utmost importance, because it plays a vital role in determining the value of W_sp_. Following the formation of the N-imp region, the gate oxide layer is grown. Subsequently, polysilicon is deposited and precisely etched back to form the gate electrode (Figure 10f). After gate electrode formation, the deposited interlayer dielectric (ILD) layer undergoes an etching and patterning process to expose the source region (Figure 10g). Finally, the source and drain electrodes are formed to complete device fabrication (Figure 10h).

Table 3 presents a comparative analysis of the key fabrication processes among different SiC TMOS devices, specifically focusing on the formation of the P-type shield region and its short connection to the source. Compared to the SiC DTMOS, SiC IETMOS and SiC MIT^2^-MOS devices, the SiC NITMOS device requires fewer fabrication steps. In addition to the SiC NITMOS device, the SiC ATMOS and Fin-SiC TMOS devices also require fewer fabrication steps. However, these devices involve the P-type ion implantation with ultra-high energy (MeV), which presents challenges in controlling the shape of the P+ shield region. The shape of the P+ shield region plays a crucial role in device performance and requires careful attention during the device fabrication.

## 5. Conclusions

In this work, a SiC TMOS device with a self-aligned N-type ion implantation technique was proposed. The impact of the W_sp_ on the device performance was comprehensively studied by numerical simulations. The presence of the P-epi layer effectively reduced the maximum electric field in the gate oxide, and the N-imp region enabled a relatively low specific on-resistance. Furthermore, the SiC NITMOS device demonstrated an improved short-circuit capability owing to the optimization of the JFET region. The gate-to-drain capacitance (C_gd_) and gate-to-drain charge (Q_gd_) of the SiC NITMOS device were significantly lower compared to the SiC DTMOS device. Such reductions are attributed to the effective screening of the coupling between the gate and drain by the P-epi layer. The superior device performance and simplicity of the manufacturing process make the proposed SiC NITMOS device highly suitable for mass production and commercialization.

## Figures and Tables

**Table 1:** Device parameters used in simulation.

Parameters	Value	Unit
N+ doping	1.0 × 10^19^	cm^–3^
N+ thickness	0.3	μm
P+ doping	2.0 × 10^18^	cm^–3^
P-well doping	1.5 × 10^17^	cm^–3^
P-well thickness	0.4	μm
CSL doping	5.0 × 10^16^	cm^–3^
CSL thickness	0.6	μm
P-epi doping	6.0 × 10^17^	cm^–3^
P-epi thickness	0.5	μm
N-imp doping	1.0 × 10^17^	cm^–3^
Trench width (TW)	1.0	μm
Trench depth (TD)	1.0	μm
Lateral spacing between trench sidewall and N-imp (W_sp_)	0–0.4	μm

**Table 2:** Summarized device performance.

	DTMOS	NITMOS (W_sp_/μm)				Unit
		0	0.1	0.2	0.3	
R_on,sp_	1.92	1.59	1.67	1.82	2.69	mΩ·cm^2^
BV	1676	1694	1696	1688	1668	V
E_ox,max_	2.62	2.56	1.90	1.20	0.57	MV/cm
BFOM ^a^	1.46	1.81	1.72	1.56	1.03	GW/cm^2^
C_gd_^b^	153	94	60	32	12	pF/cm^2^
Q_gd_	323	361	282	201	123	nC/cm^2^
Q_gd_ × R_on,sp_	620	574	471	366	331	mΩ·nC

^a^ BFOM is the value of BV^2^/R_on,sp_. ^b^ The C_gd_ is extracted at V_ds_ = 800 V.

**Table 3:** Key fabrication processes of various SiC TMOS devices.

Device	Formation of the P-Type Shield Region	Shorted to the Source (Grounding)
SiC NITMOS	1. The P-epi layer partially counter-doped by a self-aligned N-type ion implantation	—
SiC DTMOS [10]	1. The etching of the source trench 2. The vertical self-aligned P-type ion implantation into the bottom of the source trench	The titled P-type ion implantation into the sidewalls of the source trench
SiC IETMOS [11]	1. 1st P-type ion implantation in the N-type drift to form 1st P+ shield region 2. Followed by an N-type epi-layer regrowth 3. 2nd P-type ion implantation in the N-type epi-layer to form 2nd P+ shield region 4. A P-type epi-layer regrowth used as the P-well	—
SiC ATMOS [12]	1. The ultra-high energy P-type ion implantation	—
SiC MIT^2^-MOS [13]	1. The vertical self-aligned P-type ion implantation into the bottom of the gate trench	The tilted P-type ion implantation into the one side of the gate trench sidewall
Fin-SiC TMOS [14]	1. The ultra-high energy P-type ion implantation	—

“—” indicates no additional steps are needed to achieve the short connection to the source.

## Data Availability

Data are contained within the article.
